# Global vaccine coverage and childhood survival estimates: 1990–2019

**DOI:** 10.2471/BLT.23.290129

**Published:** 2024-02-29

**Authors:** Haijun Zhang, Bryan Patenaude, Haonan Zhang, Mark Jit, Hai Fang

**Affiliations:** a Department of Health Policy and Management, School of Public Health, Peking University, Beijing, China.; b International Vaccine Access Center, Bloomberg School of Public Health, Johns Hopkins University, Baltimore, United States of America.; c Department of Infectious Disease Epidemiology, London School of Hygiene & Tropical Medicine, London, England.; d China Center for Health Development Studies, Peking University, 38 Xueyuan Road, Haidian District, Beijing, 100191, China.

## Abstract

**Objective:**

To quantify the association between reduction in child mortality and routine immunization across 204 countries and territories from 1990 to 2019.

**Methods:**

We used child mortality and vaccine coverage data from the Global Burden of Disease Study. We used a modified child survival framework and applied a mixed-effects regression model to estimate the reduction in deaths in children younger than 5 years associated with eight vaccines.

**Findings:**

Between 1990 and 2019, the diphtheria–tetanus–pertussis (DTP), measles, rotavirus and *Haemophilus influenzae* type b vaccines were significantly associated with an estimated 86.9 (95% confidence interval, CI: 57.2 to 132.4) million fewer deaths in children younger than 5 years worldwide. This decrease represented a 24.2% (95% CI: 19.8 to 28.9) reduction in deaths relative to a scenario without vaccines. The DTP and measles vaccines averted 46.7 (95% CI: 30.0 to 72.7) million and 37.9 (95% CI: 25.4 to 56.8) million deaths, respectively. Of the total reduction in child mortality associated with vaccines, 84.2% (95% CI: 83.0 to 85.1) occurred in 73 countries supported by Gavi, the Vaccine Alliance, with an estimated 45.4 (95% CI: 29.8 to 69.2) million fewer deaths from 2000 to 2019. The largest reductions in deaths associated with these four vaccines were in India, China, Ethiopia, Pakistan and Bangladesh (in order of the size of reduction).

**Conclusion:**

Vaccines continue to reduce childhood mortality significantly, especially in Gavi-supported countries, emphasizing the need for increased investment in routine immunization programmes.

## Résumé

**Objectif:**

Quantifier le lien entre la baisse de la mortalité infantile et la vaccination systématique dans 204 pays et territoires entre 1990 et 2019.

**Méthodes:**

Nous avons exploité les données relatives à la mortalité infantile et à la couverture vaccinale issues de l'étude sur la charge mondiale de morbidité (GBD). Nous avons employé un cadre modifié pour la survie infantile et appliqué un modèle de régression à effets mixtes afin de mesurer l'ampleur de la diminution des décès chez les enfants de moins de cinq ans en lien avec huit vaccins.

**Résultats:**

Entre 1990 et 2019, les vaccins contre la diphtérie, le tétanos et la coqueluche (DTC), la rougeole, le rotavirus et *Haemophilus influenzae* de type b ont eu un impact significatif en permettant d'éviter près de 86,9 millions (intervalle de confiance de 95%, IC: 57,2 à 132,4) de décès à travers le monde chez les enfants de moins de cinq ans. Une baisse qui équivaut à 24,2% (IC de 95%: 19,8 à 28,9) de décès en moins par rapport à un scénario sans vaccins. Le vaccin contre la DTC a sauvé 46,7 millions de vies (IC de 95%: 30,0 à 72,7), tandis que celui contre la rougeole en a sauvé 37,9 millions (IC de 95%: 25,4 à 56,8). Les 73 pays soutenus par Gavi, l'Alliance du Vaccin, représentent 84,2% (IC de 95%: 83,0 à 85,1) du pourcentage total de réduction de la mortalité infantile grâce aux vaccins. Selon les estimations, 45,4 millions (IC de 95%: 29,8 à 69,2) de morts ont ainsi pu être évitées entre 2000 et 2019. Pour ces quatre vaccins, c'est en Inde, en Chine, en Éthiopie, au Pakistan et au Bangladesh (par ordre d'importance) que les baisses de mortalité les plus conséquentes ont été observées.

**Conclusion:**

Les vaccins continuent à exercer une influence considérable sur la réduction de la mortalité infantile, surtout dans les pays soutenus par Gavi, ce qui souligne la nécessité d'investir davantage dans les programmes de vaccination systématique.

## Resumen

**Objetivo:**

Cuantificar la asociación entre la reducción de la mortalidad infantil y la inmunización sistemática en 204 países y territorios entre 1990 y 2019.

**Métodos:**

Se utilizaron datos de mortalidad infantil y cobertura vacunal del estudio sobre la carga mundial de morbilidad. Se utilizó un marco de supervivencia infantil modificado y se aplicó un modelo de regresión de efectos mixtos para estimar la reducción de muertes en niños menores de 5 años asociada a ocho vacunas.

**Resultados:**

Entre 1990 y 2019, las vacunas contra la difteria-tétanos-tos ferina (DTP), el sarampión, el rotavirus y el *Haemophilus influenzae* tipo b se asociaron de manera significativa con un número estimado de 86,9 (intervalo de confianza del 95%, IC: 57,2 a 132,4) millones menos de muertes en niños menores de 5 años en todo el mundo. Esta disminución representó una reducción del 24,2% (IC del 95%: 19,8 a 28,9) de las muertes en relación con un escenario sin vacunas. Las vacunas DTP y contra el sarampión evitaron 46,7 (IC del 95%: 30,0 a 72,7) y 37,9 (IC del 95%: 25,4 a 56,8) millones de muertes, respectivamente. De la reducción total de la mortalidad infantil asociada a las vacunas, el 84,2% (IC del 95%: 83,0 a 85,1) se produjo en 73 países apoyados por Gavi, la Alianza para las Vacunas, con una reducción estimada de 45,4 (IC del 95%: 29,8 a 69,2) millones de muertes entre 2000 y 2019. Las mayores reducciones de muertes asociadas a estas cuatro vacunas se produjeron en India, China, Etiopía, Pakistán y Bangladesh (por orden de magnitud de la reducción).

**Conclusión:**

Las vacunas siguen reduciendo en gran medida la mortalidad infantil, sobre todo en los países que reciben apoyo de Gavi, lo que enfatiza la necesidad de aumentar la inversión en programas de inmunización sistemática.

## ملخص

**الغرض:**

تعيين الارتباط بين انخفاض معدل وفيات الأطفال، والتحصين الروتيني عبر 204 دولة وإقليم خلال الفترة من 1990 إلى 2019.

**الطريقة:**

قمنا بالاستعانة بالبيانات الخاصة بمعدل وفيات الأطفال وتغطية اللقاحات من دراسة العبء العالمي للأمراض. استخدمنا إطار عمل معدل لبقاء الطفل على قيد الحياة، وقمنا بتطبيقه على نموذج للتحوف مختلط التأثيرات، لتقدير الانخفاض في الوفيات بين الأطفال الذين تقل أعمارهم عن 5 سنوات، والمرتبطة بثمانية لقاحات.

**النتائج:**

بين عامي 1990 و2019، ارتبطت لقاحات الدفتيريا والتيتانوس والسعال الديكي (DTP)، والحصبة، وفيروس روتا، والمستدمية النزلية من النوع ب، بشكل ملموس بما يقدر بنحو 86.9 (بفاصل ثقة مقداره %95: 57.2 إلى 132.4) مليون حالة وفاة أقل بين الأطفال الأصغر من 5 أعوام حول العالم. مثّل هذا النقصان انخفاضًا بقيمة %24.2 (بفاصل ثقة مقداره %95: 19.8 إلى 28.9) في الوفيات مقارنةً بسيناريو لا يوجد به لقاحات. لقد أدت لقاح DTP والحصبة إلى تجنب 46.7 (بفاصل ثقة مقداره %95: 30.0 إلى 72.7) مليون و37.9 (بفاصل ثقة مقداره %95: 25.4 إلى 56.8) مليون حالة وفاة، على الترتيب. من إجمالي الانخفاض في معدل وفيات الأطفال المرتبطة باللقاحات، %84.2 (بفاصل ثقة مقداره %95: 83.0 إلى 85.1) حدث في 73 دولة مدعومة من جانب Gavi، التحالف العالمي لإنتاج اللقاحات، مع ما يقدر بنحو 45.4 (بفاصل ثقة مقداره %95: 29.8 إلى 69.2) مليون أقل في حالات الوفيات خلال الفترة من 2000 إلى 2019. حدث أكبر حالات الانخفاض في حالات الوفيات المرتبطة بهذه اللقاحات الأربعة في الهند، والصين، وإثيوبيا، وباكستان، وبنغلاديش (بالترتيب حسب حجم الانخفاض).

**الاستنتاج:**

تستمر اللقاحات في خفض وفيات الأطفال بشكل ملموس، وخاصة في الدول المدعومة من جانب Gavi، التحالف العالمي للقاحات والتحصين، مما يؤكد الحاجة إلى زيادة الاستثمار في برامج التحصين الروتينية.

## 摘要

**目的:**

量化 1990 至 2019 年 204 个国家和地区儿童死亡率降低与常规免疫接种之间的关系。

**方法:**

我们使用全球疾病负担研究中的儿童死亡率和疫苗覆盖率数据。我们使用了改进的儿童生存框架，并应用了一个混合效应回归模型来估计与 8 种疫苗接种相关的 5 岁以下儿童死亡人数的减少情况。

**结果:**

在 1990 年至 2019 年期间，白喉-破伤风-百日咳 (DTP)、麻疹、轮状病毒和乙型*流感嗜血杆菌*疫苗接种与全球 5 岁以下儿童死亡人数估计减少 8690 万人（95% 置信区间，CI：57.2 至 132.4）显著相关。与未接种疫苗的情况相比，这一下降表明死亡人数减少了 24.2%（95% CI：19.8 至 28.9）。DTP 和麻疹疫苗分别避免了 4670 万人（95% CI：30.0 至 72.7）和 3790 万人（95% CI：25.4 至 56.8）的死亡。在与疫苗接种相关的儿童死亡率总降幅中，84.2%（95% CI：83.0 至 85.1）发生在受全球疫苗免疫联盟所支持的 73 个国家，在 2000 至 2019 年期间死亡人数估计减少了 4540 万人（95% CI：29.8 至 69.2）。与这四种疫苗接种相关的死亡人数减少幅度最大的国家是印度、中国、埃塞俄比亚、巴基斯坦和孟加拉国（按减少幅度排序）。

**结论:**

接种疫苗可以持续显著地降低儿童死亡率，特别是在全球疫苗免疫联盟支持的国家，这表明需要增加对常规免疫接种计划的投资。

## Резюме

**Цель:**

Провести количественную оценку связи между снижением уровня детской смертности и проведением плановой иммунизации в 204 странах и территориях в 1990–2019 гг.

**Методы:**

В статье использованы данные о детской смертности и охвате вакцинацией по результатам исследования глобального бремени болезней. Для оценки снижения смертности среди детей младше 5 лет вследствие введения восьми вакцин использовалась модифицированная система оценки выживаемости детей и регрессионная модель со смешанными эффектами.

**Результаты:**

В период с 1990 по 2019 год вакцины против дифтерии-столбняка-коклюша (АКДС-вакцина), кори, ротавируса и инфекции, вызываемой бактерией *Haemophilus influenzae* типа b, в значительной степени способствовали снижению смертности среди детей младше 5 лет во всем мире на 86,9 миллиона случаев смерти (95%-й ДИ: 57,2–132,4). Это снижение представляло собой снижение смертности на 24,2% (95%-й ДИ: 19,8–28,9) по сравнению со стратегией без введения вакцин. АКДС-вакцина и вакцина против кори способствовали предотвращению 46,7 миллиона смертей (95%-й ДИ: 30,0–72,7) и 37,9 миллиона смертей (95%-й ДИ: 25,4–56,8) соответственно. 84,2% (95%-й ДИ: 83,0–85,1) от общего показателя снижения детской смертности вследствие вакцинации приходится на 73 страны, поддерживаемые Глобальным альянсом по вакцинам и иммунизации (Gavi – Global Alliance for Vaccines and Immunisation), что, по оценкам, соответствует снижению количества смертей в период 2000–2019 гг. на 45,4 миллиона случаев (95%-й ДИ: 29,8–69,2). Наибольшее снижение смертности вследствие введения этих четырех вакцин произошло в Индии, Китае, Эфиопии, Пакистане и Бангладеш (в порядке убывания).

**Вывод:**

Вакцины продолжают способствовать значительному снижению детской смертности, особенно в странах, поддерживаемых Глобальным альянсом по вакцинам и иммунизации, что подчеркивает необходимость увеличения инвестиций в программы плановой иммунизации.

## Introduction

Vaccines have substantially reduced morbidity and mortality from infectious diseases and are essential for achieving sustainable development goal (SDG) 3, which aims to ensure healthy lives and promote well-being for all at all ages.^1^^–^^3^ Vaccines are recognized as one of the most cost-effective health interventions, and play a pivotal role in enhancing global health equity by narrowing the health and financial disparities between affluent and impoverished people.^4^ Moreover, vaccines are a primary public health strategy that governments and organizations worldwide are investing in.^5^^,^^6^ Many vaccine-preventable diseases predominantly affect children younger than 5 years, including pertussis, measles and pneumococcal disease.^7^^–^^9^ The effectiveness of specific vaccines in preventing child mortality from these diseases has been well documented.^1^^,^^10^ After decades of concerted efforts, high global vaccination rates have been achieved, particularly for three doses of the diphtheria–tetanus–pertussis (DTP3) vaccine and one dose of the measles vaccine.^11^ However, in recent years, routine immunization services have faced challenges, notably the global disruption caused by the coronavirus disease 2019 (COVID-19) pandemic and persistent issues related to vaccine hesitancy.^12^^–^^14^

Different agencies have reported on the number of deaths averted due to vaccination.^15^^,^^16^ The World Health Organization (WHO) estimates that vaccines prevent 3.5–5.0 million deaths annually from diseases such as diphtheria, tetanus, pertussis, influenza and measles.^17^ The United Nations Children’s Fund (UNICEF) reports that vaccines annually save 2–3 million children from infectious diseases.^18^ Since 2000, more than 1 billion children have received full routine immunizations with the support of Gavi, the Vaccine Alliance. These immunizations are estimated to have contributed to the prevention of more than 17 million future deaths.^19^ According to Gavi’s 2023 criteria, countries are eligible for Gavi support if their most recent gross national income per capita is less than or equal to 1730 United States dollars. In 2023, 54 countries received Gavi support.^20^ Accurately estimating the cumulative impact of vaccines on the observed reduction in mortality of children younger than 5 years over time is challenging due to limitations in data; the complexity of delivering multiple concurrent vaccinations; and the increasing number of antigen-specific estimation models.^1^^,^^21^ Furthermore, since a child cannot die multiple times from different pathogens, methods are needed that can simultaneously evaluate the impact of many vaccines on child mortality.

The aim of this study was to examine the association between death in children younger than 5 years globally and vaccination with eight routine childhood vaccines, including: DTP3; one dose of measles vaccine; a complete series of rotavirus vaccine; three doses of *Haemophilus influenzae* type b (Hib3) vaccine; three doses of pneumococcal conjugate vaccine (PCV3); three doses of hepatitis B vaccine; three doses of poliomyelitis vaccine (polio vaccine); and one dose of the bacillus Calmette–Guérin (BCG) vaccine. The selection of eight childhood vaccines was primarily based on global recommendations and targeted diseases. Most of the chosen vaccines are recommended by WHO for routine immunization. This approach ensured that our study focused on vaccines that have a universally acknowledged health benefit for children. The vaccines also target diseases that are important contributors to child mortality globally. Additionally, the DTP vaccine often serves as an indicator for identifying children who have not received any vaccinations (so-called zero-dose children) and is the most widely administered vaccine globally.^11^ We calculated the reduction in the number and percentage of deaths in children younger than 5 years associated with these vaccines in each of 204 countries and territories from 1990 to 2019. We focused on 73 countries that are currently or have ever been supported by Gavi.

## Methods

### Analytic framework

In developing our estimation model, we adapted a previous analytic framework for child survival.^22^ We used data from the Global Burden of Disease Study 2019 (GBD 2019) to estimate the relationship between child mortality rates per 1000 live births and vaccine coverages rates (online repository).^23^ The model hypothesized that higher vaccine coverage rates lead to greater vaccine effectiveness, which directly lowers child mortality rates. Our model used a comprehensive approach and considered not only vaccines but also a range of determinants, such as socioeconomic, environmental and personal health factors, as control variables.

**Fig. 1:**
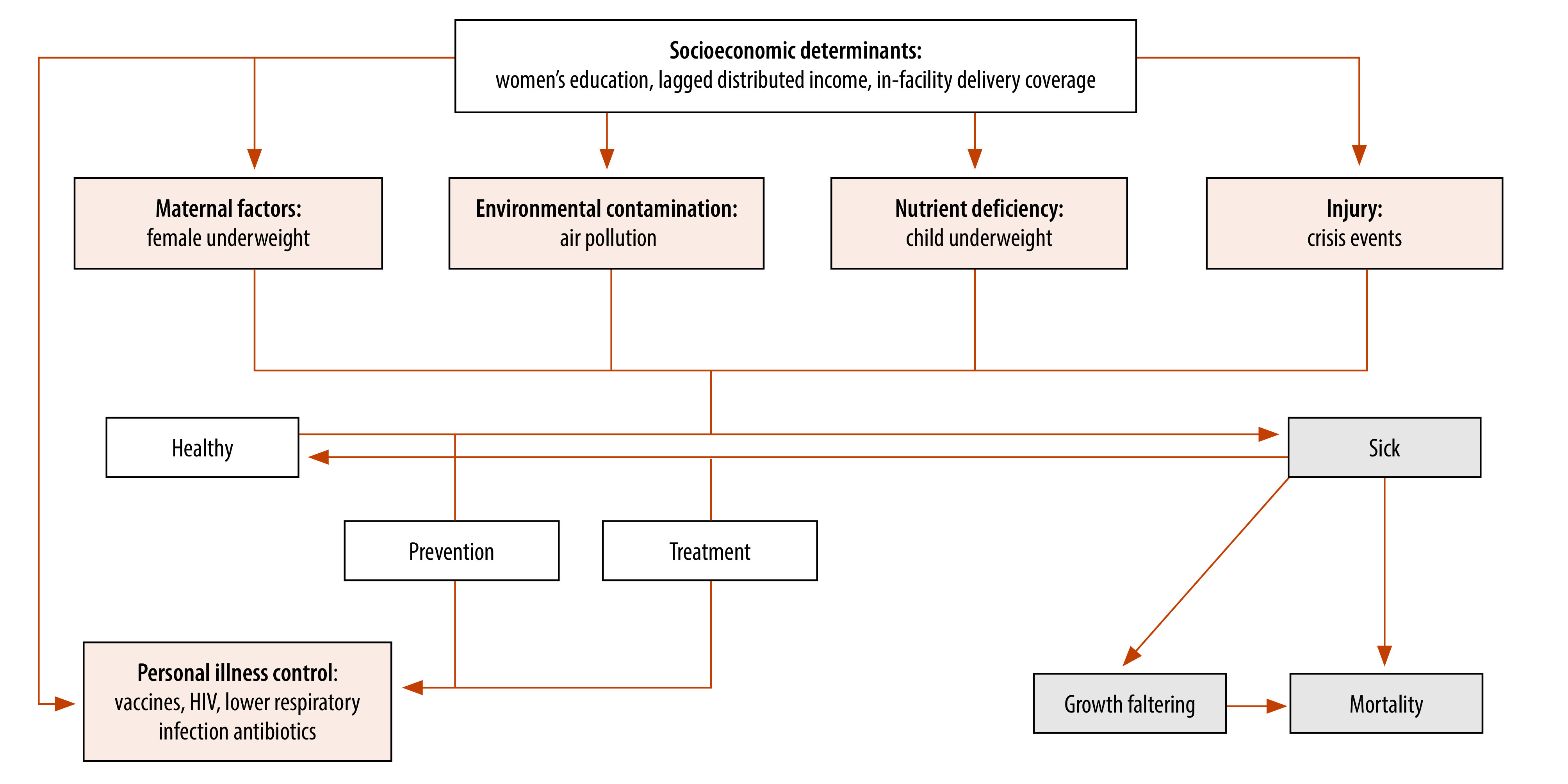
Analytic diagram for child survival

According to the above-mentioned framework, factors influencing child survival were divided into proximate determinants (intermediate variables) and socioeconomic determinants. In our model, we categorized proximate determinants into five groups: maternal factors; environmental contamination; nutrient deficiency; injury; and personal illness control. We classified socioeconomic determinants into three groups: individual level (women’s education); household level (lagged distributed income); and community level (in-facility delivery coverage). Fig. 1 illustrates these relationships.

HIV: human immunodeficiency virus.

Source: Adapted from Mosley & Chen, 2003.^22^

We took data on the coverage rates of eight childhood vaccines, mortality rates in children younger than 5 years, and the number of live births for 204 countries and territories from the GBD Study 2019, organized into seven GBD super-regions and 21 GBD regions based on geographic contiguity.^11^^,^^24^^,^^25^ Additionally, we used the online covariate database from the GBD Study 2019 to gather other important control variables. These variables represented various dimensions proposed by the above-mentioned framework,^22^ except for crisis events. In our analysis, crisis events covered five types of social and natural disasters: wars, famines, infectious disease outbreaks, earthquakes and floods (online repository).^23^ The GBD study covers more countries and territories than the member states of the United Nations, and includes areas characterized by low vaccine coverage and high childhood mortality rates. Including these areas in our analysis allows for a more comprehensive understanding of global health trends and challenges.

### Statistical model

We used a linear mixed-effects regression model, controlling for female underweight; child underweight; crisis events; antibiotics for lower respiratory infection in children; human immunodeficiency virus (HIV) infection in children; women’s education; lagged distributed income per capita; in-facility delivery coverage; and air pollution. We used the regression model to examine associations between child mortality rates and various proximate and socioeconomic factors.^24^ We included these control variables based on the analytical framework of child survival and previous models from GBD studies.^22^^,^^25^^,^^26^ The estimation model specifications are presented in the following equation and in the online repository:^23^
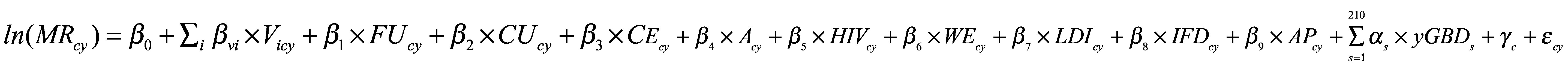
where *c* is country; *y* is year; *v* is vaccine; *i* is the i-th type of vaccines; *β *are the coefficients for vaccines, and various proximate and socioeconomic factors; *α_s_* are the coefficients for the combined year and GBD super-region fixed effect; *s* is the specific number of 210 combined years and GBD super-regions; *γ_c_* is a random effect on country; and *ε_cy_* are the random error terms. *MR* is representing mortality rate; *FU* is female underweight; *CU* is child underweight; *CE* is crisis events; *A* is antibiotics for lower respiratory infection in children; *HIV* is human immunodeficiency virus; *WE* is women's education; *LDI* is lagged distributed income per capita; *IFD* is in-facility delivery coverage; and *GBD* is Global Burden of Disease super-region.

The classification, description and data sources of the variables in this estimation model are shown in Box 1. We included eight vaccines in the model, which WHO recommends for routine immunization for all countries. Additionally, to capture the different secular trends in child mortality across geographic units, we included a combined year and GBD super-region fixed effect, namely variables related to GBD year and super-region.^25^^,^^26^

Box 1 **Variable descriptions and data sources used to calculate global vaccine coverage and childhood survival estimates, 1990–2019**

**Child mortality rate^27^**

Infant mortality rate and under-5 mortality rate. Probabilities of dying were calculated for each country and year. The natural logarithm of the child mortality rate was used as the dependent variable.

**Vaccine coverage rate^28^**

Three doses of diphtheria–tetanus–pertussis vaccine; one dose of measles-containing vaccine; complete series of rotavirus vaccine; three doses of *Haemophilus influenzae* type b vaccine; three doses of pneumococcal conjugate vaccine; three doses of hepatitis B vaccine; three doses of polio vaccine; and one dose of BCG vaccine. Estimated percentage of the population that had received specific vaccines for each country in a specific year.

**Female underweight^28^**

For women of reproductive age 10–54 years, underweight was defined differently based on age. For women younger than 20 years, underweight was defined as a BMI z-score less than −2SD. For women older than 20 years, underweight was defined as a BMI lower than 17 kg/m^2^. This measurement was applied for each country and each year.

**Child underweight^28^**

Rate of underweight (weight-for-age < –2SD from the median) in children younger than 5 years for each country in a specific year.

**Crisis events^23^**

Wars, famines, outbreaks of infectious disease, earthquakes and floods. Crisis events are represented by a binary indicator, where 1 indicates the presence of a crisis event and 0 indicates its absence, for each country each year.

**Antibiotics for lower respiratory infection in children^28^**

Percentage of children (0–5 years) with lower respiratory tract infections who were treated with antibiotics in the past 2 weeks, for each country in a specific year.

**HIV infection in children^28^**

Crude mortality rate in children associated with HIV, estimated using the improved EPP-spectrum method for the GBD 2019 study, for each country in a specific year.

**Women’s education^28^**

Average number of years of education acquired by women aged 15–49 years, for each country in a specific year.

**Lagged distributed income per capita^28^**

GDP per capita smoothed over the preceding 10 years, measured in I$ and expressed in natural logarithm for each country in a specific year.

**In-facility delivery coverage^28^**

Percentage of women giving birth in a health facility for each country in a specific year.

**Air pollution^28^**

Population-weighted average concentration of PM_2.5_, in mg/m^2^, for each country each year.

BCG: bacillus Calmette–Guérin; BMI: body mass index; EPP: estimation and projection package; GBD: Global Burden of Disease; GDP: Gross domestic product; HIV: human immunodeficiency virus; I$: international dollars; PM: particulate matter; SD standard deviation.

### Outcome measures

The primary outcomes were the reductions in the number and percentage of deaths in children younger than 5 years associated with vaccines in each country. We calculated these figures by estimating the difference in deaths in children younger than 5 years with vaccines against deaths in a hypothetical scenario without vaccines. In this counterfactual scenario, we assumed zero vaccine coverage. By comparing child deaths with and without vaccines, we estimated the reductions in child deaths associated with vaccines.

### Other analyses

We analysed three different scenarios related to the time period, infant mortality and Gavi-supported countries. For time period, we divided the 30-year span into three separate decades to assess the impact of vaccines in each period. For infant mortality, we used the rates from the GBD Study 2019 as the dependent variable in the regression model. This new model allowed us to examine the association between infant mortality and vaccines, and to identify the most influential vaccines for children younger than 1 year. We re-estimated regional models for the 73 countries supported by Gavi from 2000 to 2019 to evaluate the associations between vaccines and child deaths in these low- and middle-income countries.

The PCV vaccine was first licensed in 2000 and had lower coverage rates globally with accessibility mostly in high-income countries.^11^ We analysed data from 36 high-income countries and territories from 2000 to 2019 to assess the association between the PCV vaccine and reduction in deaths in children younger than 5 years. 

### Statistical software

We used Stata 16 (StataCorp. LP, College Station, United States of America) or R version 4.1.0 (R Foundation, Vienna, Austria) for all analyses. We considered a two-tailed *P*-value of less than 0.05 to be statistically significant for all estimated coefficients.

## Results

### Reductions in deaths

For 1990–2019, our estimate showed that four childhood vaccines (DTP3, measles, rotavirus and Hib3 vaccines) were significantly associated with a reduction in deaths globally of 86.9 (95% confidence interval, CI: 57.2 to 132.4) million in 204 countries and territories, or a global decline of 21.2 (95% CI: 14.0 to 32.3) deaths per 1000 live births (Table 1). This number represented a 24.2% (95% CI: 19.8 to 28.9) reduction compared with a counterfactual scenario without vaccines.

**Table 1:** Global reductions in deaths in children younger than 5 years associated with vaccines, 1990–2019

Vaccine	Reduction in deaths associated with vaccines (95% CI)^a^		
	No. of deaths in millions	% of deaths	Mortality rate per 1 000 live births
DTP3 vaccine	46.7 (30.0 to 72.7)	13.0 (10.4 to 15.9)	11.4 (7.3 to 17.7)
Measles vaccine	37.9 (25.4 to 56.8)	10.6 (8.8 to 12.4)	9.3 (6.2 to 13.9)
Hib3 vaccine	1.6 (1.2 to 2.0)	0.4 (0.4 to 0.4)	0.4 (0.3 to 0.5)
Rotavirus vaccine	0.7 (0.6 to 0.9)	0.2 (0.2 to 0.2)	0.2 (0.2 to 0.2)
**Total**	**86.9 (57.2 to 132.4)**	**24.2 (19.8 to 28.9)**	**21.2 (14.0 to 32.3)**

CI: confidence interval; DTP3: diphtheria–tetanus–pertussis vaccine three doses; Hib3: *Haemophilus influenzae* type b vaccine three doses.

^a^ Compared to a scenario with no vaccines.

Notes: Percentage reduction in deaths associated with vaccines = (reduction in the number of deaths associated with vaccines/estimated number of deaths without vaccines) × 100. Reduction in under-5 mortality rate per 1000 live births = (reduction in the number of deaths associated with vaccines/number of live births) × 1000.

**Fig. 2:**
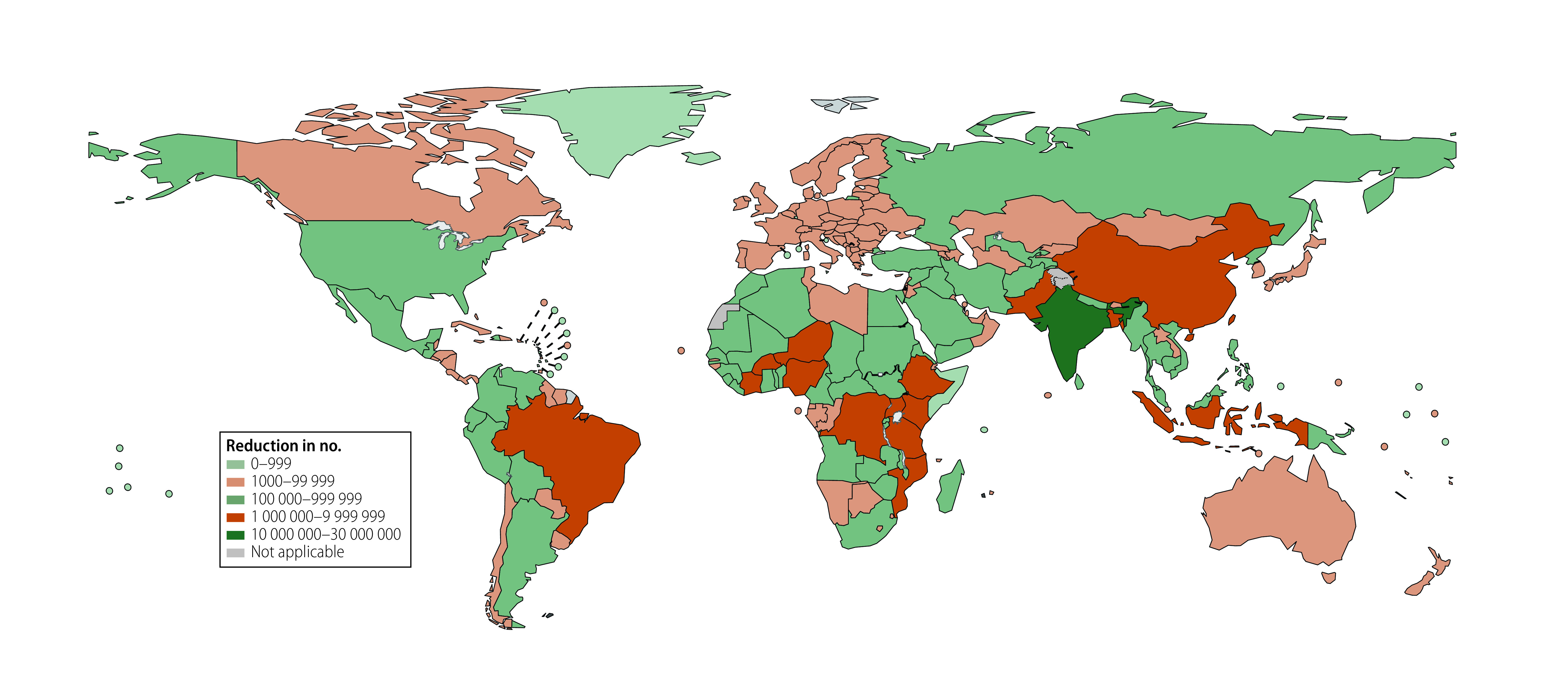
Estimated total absolute reductions in deaths in children younger than 5 years associated with four vaccines, 1990–2019

Notes: Estimates are based on data from 204 countries and areas from 1990 to 2019, comparing a scenario with no vaccines. The four vaccines are diphtheria–tetanus–pertussis vaccine three doses, *Haemophilus influenzae* type b vaccine three doses, measles vaccine one dose and rotavirus vaccine.

**Fig. 3:**
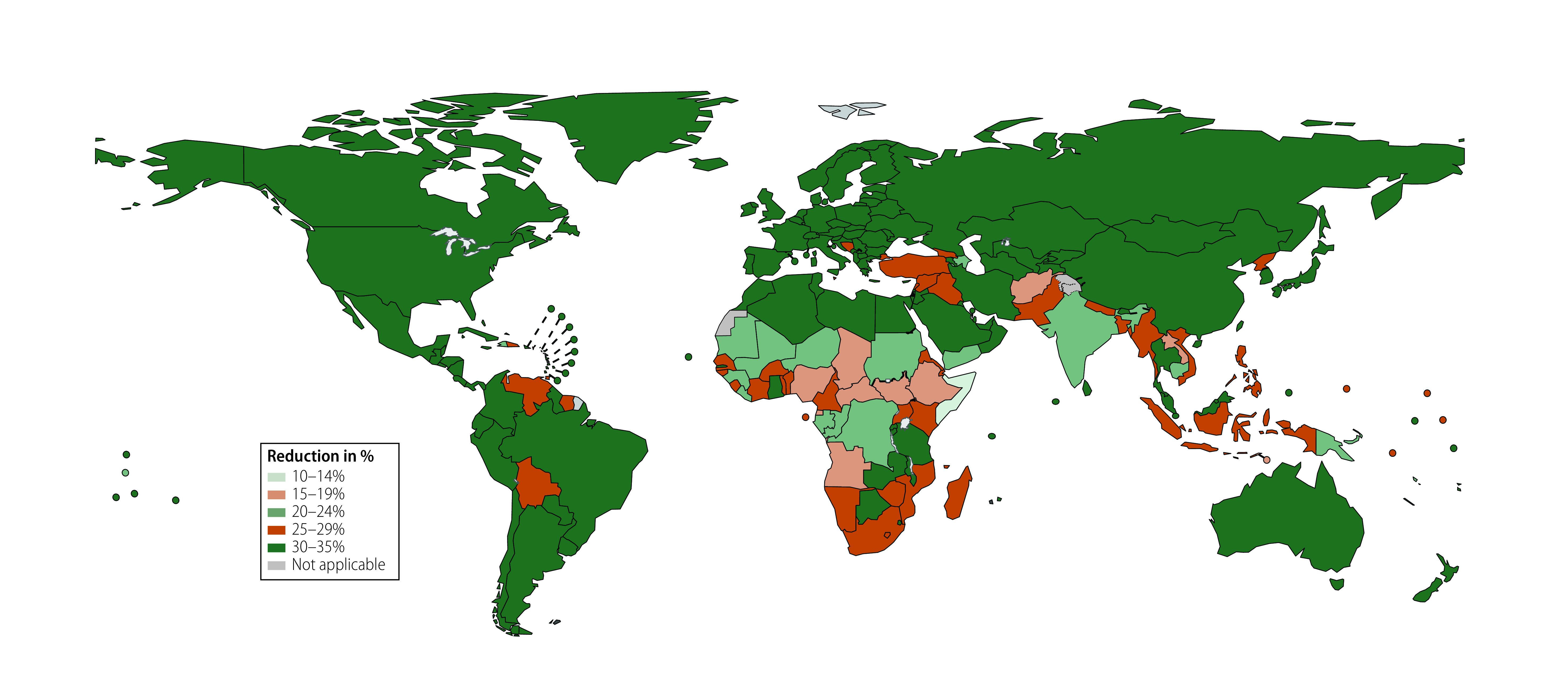
Estimated percentage reductions in deaths in children younger than 5 years associated with four vaccines, 1990–2019

Notes: Estimates are based on data from 204 countries and areas from 1990 to 2019, comparing a scenario with no vaccines. The four vaccines are diphtheria–tetanus–pertussis vaccine three doses, *Haemophilus influenzae* type b vaccine three doses, measles vaccine one dose and rotavirus vaccine.

Countries with large populations such as India, China, Ethiopia, Pakistan and Bangladesh (in order of the size of reduction) had the largest absolute reductions in deaths in children younger than 5 years associated with vaccines. Conversely, countries or areas such as Finland, Monaco, Hungary, Bermuda and Slovakia (in order of the size of reduction), which have high vaccine coverage rates, had the highest proportional reduction in deaths associated with vaccines. In contrast, countries with low vaccine coverage rates, such as Somalia, Chad, South Sudan, Afghanistan and Equatorial Guinea (in order of the size of reduction), showed the lowest proportional reductions (Fig. 2 and Fig. 3). Reductions in the number of deaths, and percentage reduction in deaths in children younger than 5 years associated with the four vaccines for all the 204 countries and territories are reported in the online repository.^23^

The reductions in deaths in children younger than 5 years associated with long-established vaccines (DTP and measles vaccines) showed a steady decline, whereas newer vaccines such as the rotavirus and Hib vaccines showed an increasing trend in their association with reducing deaths in children younger than 5 years (online repository).^23^

### Association between mortality and vaccines

In our primary estimation model, all eight vaccines were negatively and significantly associated with mortality in children younger than 5 years in the natural logarithm when each vaccine was included individually in the regression analysis without other vaccines or variables included (Table 2). When we included all eight vaccines simultaneously in the regression model without any control variables, all coefficients decreased in absolute values and the PCV3 and BCG vaccines were no longer significantly associated with reduction in deaths. When all eight vaccines were simultaneously incorporated into the estimation model together with the nine control variables, the absolute values of the vaccine coefficients decreased further. In addition, as well as the PCV3 and BCG vaccines, the polio and hepatitis B vaccines no longer showed a significant association with reduction in deaths. Only four vaccines (DTP3, measles, rotavirus and Hib3 vaccines) remained statistically significant. Overall, our model explained 91.1% of the observed variation in mortality rates in children younger than 5 years.

**Table 2:** Estimated coefficients in mixed effects linear regression models of deaths in children younger than 5 years, 1990–2019

Variable	Coefficient (95% CI)			
	Single vaccine included^a^	Eight vaccines included	Nine control variables included, but no vaccines	Eight vaccines and nine control variables included
DTP3 vaccine	−0.461 (−0.512 to −0.410)	−0.442 (−0.536 to −0.348)	NA	−0.218 (−0.299 to −0.137)
Measles vaccine	−0.409 (−0.462 to −0.356)	−0.191 (−0.258 to −0.123)	NA	−0.176 (−0.233 to −0.119)
Rotavirus vaccine	−0.081 (−0.101 to −0.060)	−0.071 (−0.092 to −0.050)	NA	−0.054 (−0.072 to −0.036)
Hib3 vaccine	−0.074 (−0.091 to –0.057)	−0.055 (−0.072 to −0.039)	NA	−0.030 (−0.044 to −0.015)
Pneumococcal conjugate vaccine, three doses	−0.034 (−0.052 to −0.015)	−0.008 (−0.027 to 0.011)	NA	−0.002 (−0.018 to 0.014)
Hepatitis B vaccine, three doses	−0.094 (−0.113 to −0.076)	−0.042 (−0.061 to −0.023)	NA	−0.002 (−0.018 to 0.014)
Polio vaccine, three doses	−0.354 (−0.408 to −0.300)	0.172 (0.079 to 0.265)	NA	0.054 (−0.026 to 0.135)
BCG vaccine	−0.084 (−0.117 to −0.052)	0.022 (−0.012 to 0.055)	NA	−0.019 (−0.009 to 0.048)
Underweight women of reproductive age	NA	NA	0.420 (0.034 to 0.807)	0.318 (−0.066 to 0.701)
Child underweight	NA	NA	10.030 (7.626 to 12.434)	7.584 (5.157 to 10.012)
Crisis events	NA	NA	0.044 (0.031 to 0.057)	0.036 (0.024 to 0.049)
Antibiotics for lower respiratory infections	NA	NA	−2.121 (−2.275 to −1.968)	−1.983 (−2.134 to −1.831)
Child HIV death rate (per 100 000 children)	NA	NA	0.002 (0.002 to 0.003)	0.002 (0.002 to 0.003)
Women's education (years)	NA	NA	−0.055 (−0.064 to −0.046)	−0.061 (−0.070 to −0.052)
Lagged distributed income (US$)	NA	NA	−0.081 (−0.100 to −0.062)	−0.096 (−0.114 to −0.077)
In-facility delivery, proportion	NA	NA	−0.310 (−0.369 to −0.251)	−0.249 (−0.308 to −0.190)
Air pollution (PM_2.5_)	NA	NA	−0.001 (−0.003 to 0.000)	−0.001 (−0.003 to 0.000)

BCG: bacillus Calmette–Guérin; CI: confidence interval; DTP3: diphtheria–tetanus–pertussis vaccine three doses; Hib3: *Haemophilus influenzae* type b vaccine three doses; HIV: human immunodeficiency virus; NA: not applicable; PM: particulate matter; US$: United States dollars.

^a^ We constructed separate models for each vaccine.

Additionally, the associations with the nine control variables were consistent with our expectations (Table 2). Child underweight, crisis events, antibiotics for lower respiratory infection, child HIV death rate, women’s education, lagged distributed income and in-facility delivery coverage were all significantly associated with deaths in children younger than 5 years. Air pollution and underweight in women of reproductive age were not significantly associated with child mortality.

### Other analyses

**Fig. 4:**
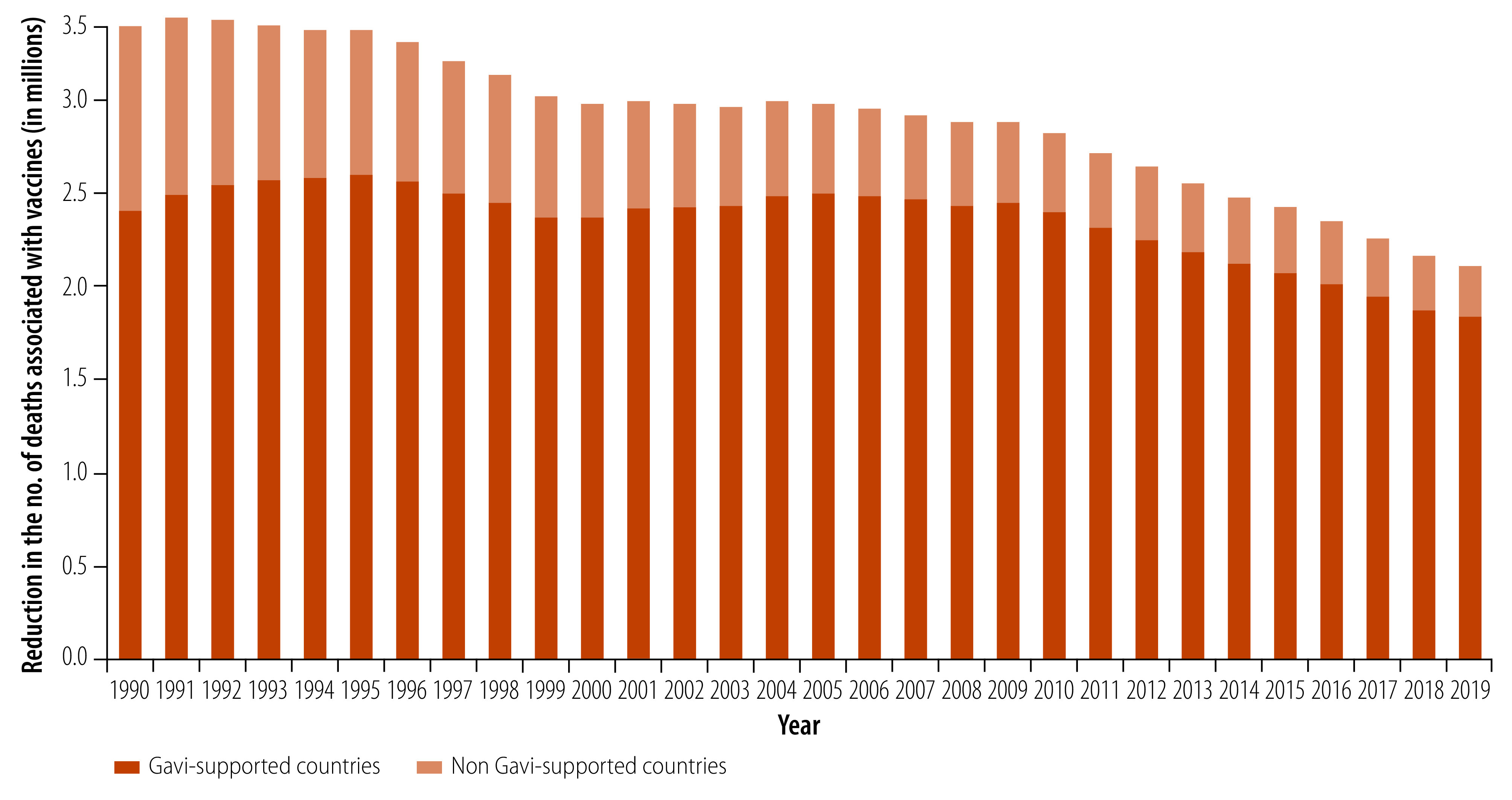
Estimated reductions in the number of deaths in children younger than 5 years associated with four vaccines in countries and territories with and without Gavi support, 1990–2019

In the 73 Gavi-supported countries, vaccines were linked to a reduction of 45.4 (95% CI: 29.8 to 69.2) million deaths between 2000 and 2019, with the DTP3 vaccine associated with the largest reduction (52.2%). Most of the reductions in child deaths (84.2%, 95% CI: 83.0 to 85.1) were associated with vaccines administered in low- and middle-income countries, as most of these countries have received Gavi support since 2000 (Fig. 4 and online repository).^23^

Gavi: Gavi, the Vaccine Alliance.

Notes: Estimates are based on data from 204 countries and territories from 1990 to 2019, comparing a scenario with no vaccines. The four vaccines are diphtheria–tetanus–pertussis vaccine three doses, *Haemophilus influenzae* type b vaccine three doses, measles vaccine one dose and rotavirus vaccine.

The DTP3 vaccine was significantly associated with reductions in deaths in children younger than 5 years across all time periods from 1990 to 2019 (online repository).^23^ In contrast, the measles vaccine was only associated with reductions in deaths children younger than 5 years during the decade 1990–1999 (online repository).^23^ The absolute reduction in deaths associated with the DTP3 and measles vaccines decreased slightly over the three decades. Newer vaccines, such as rotavirus and Hib, were associated with mortality reductions after 2000 (online repository).^23^

The regression results for infant deaths were similar to those for deaths in children younger than 5 years (online repository).^23^ A total reduction of 45.2 (95% CI: 29.0 to 70.5) million infant deaths, or an 11.0% (95% CI: 7.1 to 17.2) decrease in infant mortality, was associated with the four vaccines. The measles vaccine was associated with the largest decrease in infant deaths (11.3%; online repository).^23^ From 2000 to 2019, the four vaccines were associated with 23.9 (95% CI: 15.2 to 37.3) million averted infant deaths in 73 Gavi-supported countries.

The results of herd immunity scenario analyses are reported in the online repository.^23^ The robustness of the regression results was maintained even after dropping all statistically insignificant variables (online repository).^23^

The estimated coefficient for PCV3 in 36 high-income countries was statistically significant (−0.081, 95% CI: –0.111 to −0.052) and larger than the coefficients for the rotavirus (−0.059, 95% CI: –0.092 to −0.027) and Hib3 (−0.029, 95% CI: –0.057 to −0.002) vaccines (online repository).^23^

## Discussion

From 1990 to 2019, vaccines were significantly associated with the reduction in deaths in children younger than 5 years. Most of this reduction occurred in Gavi-supported countries, and was attributed to rapid increases in coverage rates and the high mortality due to vaccine-preventable diseases in these countries. Long-established vaccines, such as the DTP and measles vaccines, continued to be effective in preventing child deaths. Since 2000, the rotavirus, Hib and PCV vaccines have contributed increasingly to reducing deaths in children younger than 5 years. Our model predicted that a hypothetical 10% average decrease in the global coverage rates of DTP3, measles, rotavirus and Hib3 vaccines from 1990 to 2019 would have resulted in 4.7 (95% CI: 3.0 to 7.3), 3.8 (95% CI: 2.5 to 5.7), 0.2 (95% CI: 0.1 to 0.2) and 0.07 (95% CI: 0.06 to 0.09) million fewer deaths averted, respectively, in children younger than 5 years.

While our results align those of previous studies, our estimates of deaths averted are slightly lower, which is likely due to the simultaneous consideration of competing risk factors. For example, the decrease in absolute values for vaccines when we included all eight vaccines simultaneously implies that the bivariate estimates of the association between specific vaccine coverage and under-five deaths might be overestimates due to double-counting. The Vaccine Impact Modelling Consortium estimated that three vaccines (measles, rotavirus and Hib3) averted 30.7 million deaths in children younger than 5 years, while the same vaccines in our study were associated with a reduction of 23.6 million deaths in the same 97 low- and middle-income countries (one of the 98 countries was not comparable).^1^ In a 2018 study, the Hib3 vaccine was estimated to have averted 1.2 million deaths in children younger than 5 years globally from 2000 to 2015,^7^ while our estimate was about 1.04 million deaths averted. Similarly, another study estimated that the measles, rotavirus and Hib3 vaccines averted about 12.5 million deaths in 73 countries supported by Gavi between 2011 and 2020,^21^ whereas our figure was 10.3 million. These differences might also stem from the use of different data sources, assumptions and models in these three studies.

In our main regression model with 204 countries and territories between 1990 and 2019, four vaccines (PCV3, polio, BCG and hepatitis B3) were not significantly associated with deaths in children younger than 5 years. Although the PCV vaccine is highly efficacious, two main factors explain this finding. First, the high cost of PCV made it difficult to increase global vaccination rates, particularly in countries not supported by Gavi.^21^^,^^29^^,^^30^ Second, PCV was first licensed in 2000 in the United States of America, and the Pneumococcal Advanced Market Commitment, which facilitated global roll-out of this vaccine, only started in 2009.^31^ Therefore, the impact of the PCV vaccine was likely minimal for most of the years covered in our 30-year model. Improved effectiveness of PCV has been observed in recent years, predominantly in high-income settings. The finding that polio, BCG and hepatitis B vaccines were not associated with significant reductions in death is likely due to the underlying disease prevalence, incidence or time-to-death after infection. For instance, the poliovirus has been endemic in only two countries since 2017, Afghanistan and Pakistan.^32^ Therefore, in most parts of the world, regular transmission of the poliovirus was virtually absent and hence administration of the polio vaccine would not correlate with child mortality. Rather, the polio vaccine serves to prevent the recurrence of the disease in countries where polio has already been eradicated. Tuberculosis and hepatitis B, which are targeted by the BCG and hepatitis B vaccines, respectively, primarily cause deaths in adults.^1^^,^^32^

Our study has several limitations. First, despite the GBD study’s systematic approach to gathering data on deaths in children younger than 5 years and infants, vaccine coverage and other important control variables, it might not have captured all available data.^25^^,^^26^ Second, some unobserved confounding may have occurred in causal inferences using ecological and cross-national databases. A mixed-effects model might not fully account for all possible biases, leading to potential endogeneity in the model.^33^^,^^34^ Third, many vaccines also significantly affect child morbidity and household health spending by preventing severe but non-fatal disease episodes. Therefore, our estimates did not fully represent the broader benefits of improving vaccine coverage. Finally, projecting the future impact of vaccines was not feasible in our historical estimation model due to uncertainties in future trends in vaccine coverage and covariates, especially in the context of disruptions caused by the COVID-19 pandemic.^35^

In summary, this study demonstrates that a significant portion of the reduction in childhood mortality over three decades was associated with global improvements in routine immunization coverage. This decrease was particularly evident in Gavi-supported countries, where most of the reductions in under-five mortality attributable to vaccines were observed. Despite the complex and interrelated determinants of child mortality, vaccination is still a high priority and brings high returns on investment. Maintaining high coverage rates in routine immunization programmes, even for long-established vaccines such as DTP and measles, as well as increasing the availability of rotavirus, Hib and PCV vaccines, is important to achieving the Immunization Agenda 2030 and SDG target 3.2, that is to end preventable deaths of newborns and children younger than 5 years.^2^
