# Corrigendum

**DOI:** 10.2471/BLT.24.100524

**Published:** 2024-05-01

In: Zhang H, Patenaude B, Zhang H, Jit M, Fang H. Global vaccine coverage and childhood survival estimates: 1990–2019. Bull World Health Organ. 2024 Apr 1;102(4):276–87, 

on page 282, the second sentence in the second paragraph should read as follows:

A total reduction of 45.2 (95% CI: 29.0 to 70.5) million infant deaths, or an 19.7% (95% CI: 15.6 to 24.2) decrease in infant mortality, was associated with the four vaccines.

